# Pancreatic cancer: Surgery is a feasible therapeutic option for elderly patients

**DOI:** 10.1186/1477-7819-9-10

**Published:** 2011-01-27

**Authors:** Guy Lahat, Ronen Sever, Nir Lubezky, Ido Nachmany, Fabian Gerstenhaber, Menahem Ben-Haim, Richard Nakache, Josef Koriansky, Josef M Klausner

**Affiliations:** 1 Department of Surgery at The Sourasky Medical, Tel-Aviv, Israel

## Abstract

**Background:**

Compromised physiological reserve, comorbidities, and the natural history of pancreatic cancer may deny pancreatic resection from elderly patients. We evaluated outcomes of elderly patients amenable to pancreatic surgery.

**Methods:**

The medical records of all patients who underwent pancreatic resection at our institution (1995-2007) were retrospectively reviewed. Patient, tumor, and outcomes characteristics in elderly patients aged ≥ 70 years were compared to a younger cohort (<70y).

**Results:**

Of 460 patients who had surgery for pancreatic neoplasm, 166 (36%) aged ≥ 70y. Compared to patients < 70y (n = 294), elderly patients had more associated comorbidities; 72% vs. 43% (p = 0.01) and a higher rate of malignant pathologies; 73% vs. 59% (p = 0.002). Operative time and blood products consumption were comparable; however, elderly patients had more post-operative complications (41% vs. 29%; p = 0.01), longer hospital stay (26.2 vs. 19.7 days; p < 0.0001), and a higher incidence of peri-operative mortality (5.4% vs. 1.4%; p = 0.01). Multivariable analysis identified age ≥ 70y as an independent predictor of shorter disease-specific survival (DSS) among patients who had surgery for pancreatic adenocarcinoma (n = 224). Median DSS for patients aged ≥ 70y vs. < 70y were 15 months (SE: 1.6) vs. 20 months (SE: 3.4), respectively (p = 0.05). One, two, and 5-Y DSS rates for the cohort of elderly patients were 58%, 36% and 23%, respectively.

**Conclusions:**

Properly selected elderly patients can undergo pancreatic resection with acceptable post-operative morbidity and mortality rates. Long term survival is achievable even in the presence of adenocarcinoma and therefore surgery should be seriously considered in these patients.

## Introduction

Pancreatic cancer is the fourth leading cause of cancer related mortality in the U.S. with an estimated five-year survival of about 5% reflecting the aggressive nature of this disease [1]. In spite of recent advances in the fields of medical oncology and radiation, and the common use of neo-adjuvant and/or adjuvant systemic chemotherapy complete resection (R0) is the single most important factor determining outcome [2,3]. Unfortunately, the majority of patients are deemed unresectable at the time of diagnosis due to distant metastasis or a locally extensive disease [3]. Historically, peri-operative mortality and post-operative complications rates following pancreatic surgery were unacceptable [4]; however, during the last two decades surgical outcome significantly improved with reported data showing a peri-operative mortality rate of less than 2% in high volume centers [5,6]. Nevertheless, pancreatic surgery is still widely considered as a complex procedure which is associated with considerable morbidity and mortality [7] and therefore clinicians still fail to refer patients with early stage pancreatic cancer to surgery [8].

As a result of demographic changes in developed countries, the proportion of the elderly population is rapidly increasing; by 2025, 20% of Americans will be 65 years or older as compared with 12% of the current population [9]. Since the incidence of cancer increases with aging, the burden of cancer is likely to increase as well [10]; consequently, the number of elderly patients diagnosed and treated for pancreatic cancer is also expected to increase. The rising number of elderly patients diagnosed with pancreatic cancer creates a dilemma for gastroenterologists, medical oncologists, and surgeons who might hesitate to treat these patients in a comparably manner as used for younger patients due to factors such as decreased performance status, associated comorbidities, and the natural history of the disease.

Over the last decade several reports described outcome for pancreatic surgery in elderly patients [4,6,11-15]; however, results are inconsistent. We utilized a relatively large database of 475 pancreatectomies, seeking to evaluate early and late surgical outcome in elderly pancreatic cancer patients; in addition, we examined whether age per-se is a risk factor for adenocarcinoma-specific mortality following complete resection.

## Methods

The study was approved by our institutional review board; a waiver of consent was granted for the proposed patient record review. The medical records of all patients (n = 475) who had surgery for pancreatic neoplasm from January 1995 to April 2007 were evaluated. Patients with metastatic disease and/or incomplete data were excluded (n = 15); 460 patients were included in the study cohort. All patients remained in active clinical follow-up through our out-patient clinic.

Evaluation methods for clinical determinations of interest included various radiographic (CT, MRI, PET, US, etc.) and clinical examinations. Some patients were treated with systemic chemotherapy and/or radiation in accordance with the physician recommendations of the multidisciplinary planning conference. The recommendations for surgical, chemotherapeutic, and radiation treatments were based on an evaluation of clinical prognostic factors.

Clinical, imaging and pathological data of all patients were retrospectively reviewed. Outcomes were compared between two patients groups according to age: under 70 years (n = 294) vs. 70 years or older (n = 166) and the type of operation performed: Pancreaticoduodenectomy (PD) vs. other pancreatectomies (Distal subtotal pancreatectomy, total pancreatectomy and enucleation). Surgical technique was standardized with systematic lymphadenectomy, patients who had partial resection of superior mesenteric vein and/or portal vein were included in the study cohort. None of the elderly patients included in the study cohort had pyloric preservation PD; the vast majority of the younger cohort had their pylorus preserved.

Demographic characteristics, comorbidities, intraoperative data, pathologic data, perioperative morbidity, perioperative mortality (30-day) and survival were compared between both age groups.

The following preoperative clinicopathological features were included in the analysis: gender, age, clinical presentation, and imaging findings. All patients were evaluated by imaging studies prior to surgery including ultrasound (US), computed tomography (CT) and endoscopic ultrasound (EUS). Endoscopic retrograde cholangiopancreaticography (ERCP) was performed in the minority of patients with pancreatic head tumors, usually as a draining procedure. Associated comorbidities were documented and categorized as ischemic heart disease, diabetes Melitus, hypertension, CVA/TIA, second primary malignancies, and others. Intraoperative findings that were evaluated included type of pancreatic resection, tumor location, tumor size, number of packed cells (PC) units consumed and length of operation. Overall incidence of postoperative complications was reviewed; the following peri-operative complications were documented and included in the present analysis: massive post-operative bleeding, septic complications, renal failure, pancreatic fistula, thromboembolic event, and evisceration. Pancreatic fistula was documented in the occurrence of >30 ml amylase rich fluid from drains on postoperative day seven or upon discharge with surgical drains in place regardless of the amount. All other complications were categorized as others. Peri-operative mortality was defined as in-hospital death within 30 days after surgery. Data concerning microscopic margins of resection, histological type, tumor grade, and lymph node involvement were obtained from the pathological reports.

### Statistical analysis

The endpoint of this study was disease- specific survival. Disease-specific survival (DSS) was calculated as the elapsed time from operation at our institution to death from disease; data were censored at time of last follow-up.

Patients who died from other causes or unknown causes were included in the DSS analysis as censored cases. Kaplan-Meier [16] curves were constructed to determine DSS time. The log-rank test [17] was used to compare DSS between subgroups of patients. Comparison between patient groups with regard to demographics and clinical variables was performed using Mann-Whitney and Chi-square test as applicable. All values are expressed as mean ± SD for parametric and median for non parametric variables. Univariable Cox [18] proportional hazards regression models were examined to assess the ability of patient characteristics to predict DSS. A multivariable Cox model was performed using backward elimination with p-value cutoff of 0.05. All computations were carried out in SPSS for windows software version 17.0.

## Results

### Patient and tumor characteristics

One hundred and sixty-six patients (36%) over the age of 70 years who had surgery for pancreatic neoplasm at our institution were identified and are included in the elderly study cohort. Patients and tumor characteristics are depicted in Table 1. The median age at the time of presentation to our institution was 75 years (range, 70-87); there were 64 men (39%) and 102 women (61%). One hundred and ten patients (66%) had associated comordities with an American Society of Anesthesiologists (ASA) score of 3, 10 patients (6%) were categorized as ASA score 4; cardiovascular comorbidities (ischemic heart disease, previous myocardial infarction, hypertension, and previous cerebrovascular accident) accounted for 87% of the total documented conditions, whereas significant respiratory or renal insufficienty accounted for less than 10%. One hundred and fifty-eight patients (95%) had surgery for malignant or potentially malignant tumors (e.g., Intra ductal papillay mucinous tumor). One hundred and twenty-four tumors (73%) were located in the head of the pancreas, and the most common histology was pancreatic ductal adenocarcinoma (45%, n = 75).

**Table 1:** Clinical and pathological data

	All patients N = 460	<70 years N = 294	≥**70 years** N = 166	P value
**Age**, years, median (range)	65 (19-87)	55 (19-69)	75 (70-87)	**<0.0001**

**Gender**				
Male (%)	214 (47%)	150 (51%)	64 (39%)	**0.01**
Female (%)	246 (53%)	144 (49%)	102 (61%)	

ASA* score				
1/2	195 (42%)	156 (53%)	39 (24%)	**0.002**
≥ 3	246 (53%)	126 (43%)	120 (72%)	
Unknown	19 (5%)	12 (4%)	7 (4%)	

**Second primary cancer **(per history)	51 (11%)	25 (9%)	26 (16%)	**0.02**

**Jaundice**	160 (35%)	83 (28%)	77 (46%)	**<0.0001**

**Diagnostic evaluation**				
Tomography	443 (96%)	285 (97%)	158 (95%)	0.5
Ultrasound	149 (32%)	99 (34%)	50 (30%)	0.37
Endoscopic ultrasound	291 (63%)	197 (67%)	94 (67%)	0.73
ERCP**	140 (30%)	85 (29%)	55 (33%)	0.21

**Preoperative anemia **(Hgb < 11 g/dL)	38 (8%)	17 (6%)	21 (13%)	0.12

**Tumor site**				
Head	297 (65%)	173 (59%)	124 (73%)	**0.004**
Body/tail	163 (35%)	121 (41%)	42 (27%)	

**Invasive cancer**	293 (64%)	173 (59%)	121 (73%)	**0.002**

**Histology**				
Ductal adenocarcinoma	180 (39%)	105 (36%)	75 (45%)	0.2
Papillary carcinoma	44 (10%)	24 (8%)	20 (12%)	0.41
Cholangiocarcinoma	20 (4%)	14 (5%)	6 (4%)	0.38
IPMN	47(10%)	25 (9%)	22 (13%)	0.16
MCN	53 (12%)	35 (12%)	18 (11%)	0.36
NET	33 (7%)	28 (10%)	5 (3%)	**0.009**
Others	83 (18%)	63 (20%)	20 (12%)	0.1
				

*ASA- American society of anesthesiologists; **ERCP- Endoscopic retrograde cholangiopancreatography

### Treatment characteristics

All 166 patients included in the study cohort had complete macroscopic resection at our institution. Table 2 depicts treatment rendered. One hundred and twenty patients (72%) had pancreaticoduodenectomy (PD), 42 (25%) underwent distal pancreatectomy, two (1.5%) patients had total pancreatectomy, and two (1.5%) had their tumors enucleated. Vascular resections were performed in three cases. Mean operative time for patients who had PD and distal pancreatectomy was 318 minutes (SE: 67) and 179 minutes (SE: 80), respectively. Mean number of packed cells units consumed during surgery for the entire cohort was 1.74 (SE: 2.2); 104 patients (63%) did not receive blood products during their operation. Microscopic margins were negative in 95% of malignant tumor cases (n = 158). 25 patients (15%) received systemic chemotherapy in conjunction with surgery, the vast majority (92%; n = 23) in the post-operative setting.

**Table 2:** Treatment characteristics

	All patients N = 460	<70 years N = 294	≥**70 years** N = 166	P value
**Surgery**				
Pancreaticoduodenectomy (PD)	293 (64%)	173 (59%)	120 (72%)	**0.004**
Distal pancreatectomy	147 (32%)	105 (35%)	42 (25%)	**0.03**
Total pancreatectomy	16 (2%)	14 (5%)	2 (1.5%)	**0.05**
Enucleation	4 (1%)	2 (1%)	2 (1.5%)	0.29

**Mean operative time **(minutes)*				
PD	282 (SE:72)	268 (SE:55)	318 (SE:67)	0.07
Distal pancreatectomy	160 (SE:97)	155 (SE:92)	179 (SE:80)	0.13

Intra-operative PC** consumption				
Mean (# PC units)	1.72 (SE:2.5)	1.69 (SE:1.7)	1.74 (SE:2.2)	0.42
				
**Chemotherapy**	225 (49%)	200 (68%)	25 (15%)	**0.003**
				

*All patients who had total pancreatectomy/enucleation were excluded from mean operative time analysis; **PC- Packed cells

### Early post-operative outcome

There were no cases of intra-operative deaths; mean post-operative stay for patients who underwent PD and distal pancreatectomy was 28.2 days (SE: 15.3) and 18.7 days (SE: 11.7), respectively. The 30-day post-operative mortality rate for the entire cohort was 5.4% (n = 9); 8 out of 120 patients (6.6%) had PD, one out of 43 patients underwent distal pancreatectomy (2.3%). Five patients developed multi organ failure following septic complications, three died due to pulmonary embolism, and one due to hemorrhagic shock followed by acute myocardial infarction and multi organ failure. Overall complication rate was 41% (n = 68); 28 (17%) of them were defined as medical, whereas 40 (24%) were surgical. The most common were septic complications (31.3%; n = 52), 18 (11%) of these patients were diagnosed with pneumonia; pancreatic fistula (12%; n = 20). Most surgical complications were approached in a conservative manner; however, 10 patients (6%) underwent re-operation, mostly due to post-operative bleeding (n = 6).

### Long-term outcome

**Figure 1:**
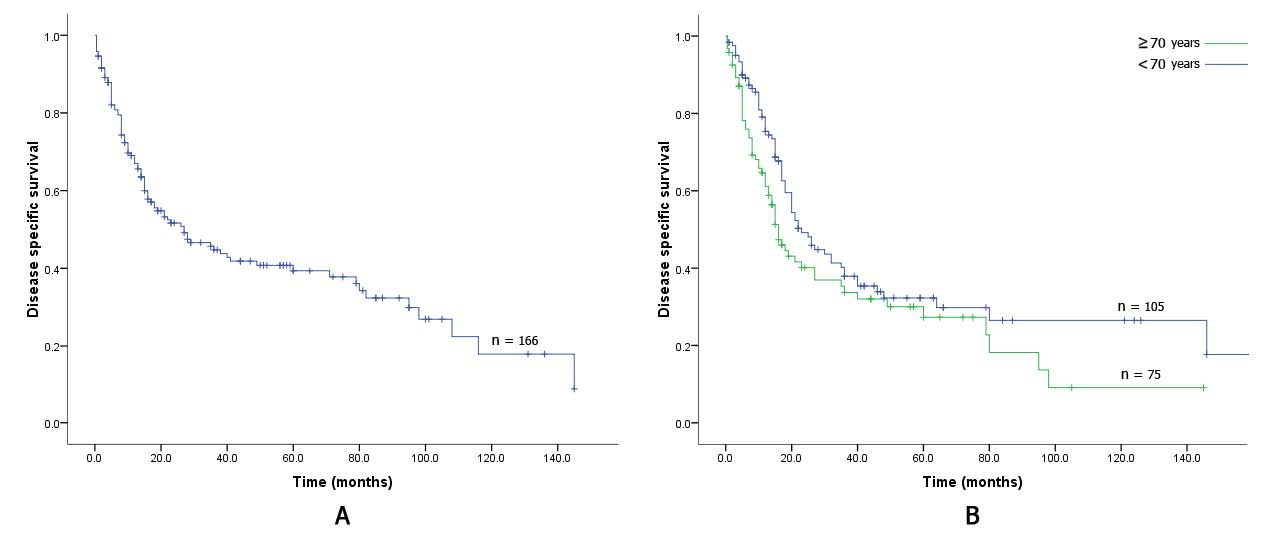
**Kaplan-Meier curves for disease-specific survival for the whole cohort of elderly patients (A), and for ductal adenocarcinoma elderly patients stratified for ductal adenocarcinoma patients aged <70 years (blue line) versus ≥70 years (B)**.

All patients were followed in the out patient clinic at our institution and the median follow-up interval for the entire cohort and for the subset of survivors was 22 months (range, 1-187) and 41.5 months (range, 3-187), respectively. Overall, 92 patients (55.4%) died of disease; Kaplan-Meier survival analysis was performed for the entire cohort and for patients treated for pancreatic adenocarcinoma, separately. The estimated median survival for the entire cohort was 27 months (95% CI: 17-43) with 1-, 2-, and 5-year DSS rate of 67% (SE: 2), 52% (SE: 2.4), and 40% (SE: 2.7), respectively (Figure 1A). The estimated median survival for the sub-cohort of elderly patients who were surgically treated for pancreatic adenocarcinoma (n = 75) was 15 months (95% CI: 12.5-26.5) with 1-, 2- and 5-year DSS rate of 58% (SE: 3.1), 36% (SE: 3.6), and 23% (SE: 3.7), respectively (Figure 1B).

Evaluating whether age ≥ 70 years is a risk factor for shorter DSS, we performed univariable Cox regression analyses for all patients who had surgery for pancreatic ductal adenocarcinoma (n = 180) at our institution. Univariable analysis identified age 70 years or older, tumor diameter larger than 3 cm, positive microscopic margins, poorly differentiated histology, and lymphatic metastasis emerged as significant predictors of decreased DSS.

In the multivariable analysis, age ≥ 70 years (hazard ratio [HR] = 1.64; 95% CI:1.16-3.45), tumor size ≥ 3 cm (HR = 1.42; 95% CI:1.21-2.87), poorly differentiated histology (HR = 1.87; 95% CI:1.07-3.19), and lymphatic metastasis (HR = 2.13; 95% CI:1.48-3.6) remained independent prognosticators of adverse outcome (Table 3).

**Table 3:** Multivariable Cox proportional hazards models for pancreatic ductal adenocarcinoma- specific survival

Variable	Levels	HR (95% CI)
Age	≥70 years vs. <70 years	1.64 (1.16-3.45)
Tumor size	>3 cm vs. ≤3 cm	1.42 (1.21-2.87)
Differentiation	Poorly vs. well	1.87 (1.07-3.19)
Lymphatic metastasis	Positive vs. negative	2.13 (1.48-3.61)

### Intergroup age-related comparisons

Next, we analyzed a cohort of 460 patients who had surgery for pancreatic neoplasm at our institution between 1995-2007 trying to identify clinical, pathological, treatment, and outcome differences between two patient sub-groups of age, ≥70 years (n = 166) vs. <70 years (n = 294; Table 1).

Comparing patients characteristics, gender, incidence of associated comorbidities, and jaundice at the time of diagnosis differed significantly between the two age groups (Table 1); 64 patients (39%) aged 70 years or older were men vs. 150 (51%) in the younger cohort (p = 0.01). 120 patients ≥70 years (72%) had associated comorbidities vs. 126 (43%) patients <70 years (p = 0.002). Jaundice, as a presenting sign, was more common in the older subset of patients, 46% (n = 77) vs. 28% (n = 83) in the younger group (p < 0.0001). Comparing tumor characteristics, pancreatic head location was more common in patients ≥70 years, 72% (n = 120) vs. 59% (n = 173) in patients younger than 70 years (p = 0.004). A higher rate of malignant histologies was identified in patients aged 70 years or older vs. patients younger than 70 years; 73% (n = 121) vs. 59% (n = 173; p = 0.002), respectively. Neuroendocrine tumors were less frequent in elderly patients 3% (n = 5) vs. 10% (n = 28) in the younger cohort (p = 0.009). Among adenocarcinoma patients tumor size, its differentiation, and lymph node involvement were comparable between the two groups.

Pre-operative workup did not significantly differ between the two sub-cohorts; these include abdominal CT which was performed in more than 95% in both groups, EUS and EUS guided biopsy/FNA, and ERCP (Table 1). Comparison of treatment variables demonstrated that the rate of elderly patients treated with adjuvant chemotherapy was lower in comparison to the younger sub-cohort; 15% vs. 68%, respectively (p = 0.003). No other significant treatment differences were identified; nevertheless, comparing patients who underwent PD, there was a trend towards a prolonged mean operative time among the elderly vs. the younger age group, 318 (SE:67) minutes vs. 268 minutes (SE:55), respectively (p = 0.07). Estimated mean intra-operative blood loss did not differ.

Outcomes characteristics are depicted in Table 4. Since most elderly patients underwent PD, which is considered a more hazardous operation, we separately analyzed early post-operative outcome for this sub-cohort of patients (n = 293). Mean post-operative length of stay was 28.2 days in the group of patients older than 70 years (range, 10-63) vs. 19.7 (range, 7-55) days in the younger age cohort of patients (p < 0.0001). The incidence of post-operative complications in patients older than 70 years was 41% vs. 29% in patients younger than 70 years (p = 0.01). Septic complications and renal failure, in particular, were more common in patients older than 70 years, 31% vs. 21% (p = 0.02), and 8% vs. 1.5% (p < 0.0001) in the younger cohort, respectively. Pancreatic fistula occurred in 12 (20%) vs. 20 (8%) patients aged 70 years or older vs. patients younger than 70 years, respectively (p = 0.12). Post-operative mortality, defined as death within 30 days from surgery, was higher in the elderly population, 5.8% (n = 7) vs. 2.3% (n = 4) in patients ≥70 years vs. < 70 years, respectively (p = 0.02).

**Table 4:** Outcomes characteristics

	All patients N = 460	<70 years N = 294	≥**70 years** N = 166	P value
**Mean hospital stay (days)**				
Overall	18.6 (SE:17.5)	16.8 (SE:12.1)	26.2 (SE:16.4)	**<0.0001**
PD*	23 (SE:18.1)	19.7 (SE:14.8)	28.2 (SE:15.3)	**<0.0001**

**Postoperative mortality (30 days)**				
Overall	13 (2.8%)	4 (1.4%)	9 (5.4%)	**0.05**
PD*	11 (3.7%)	4 (2.3%)	7 (5.8%)	**0.02**

Postoperative (PD*) complications				
Overall	153 (33%)	85 (29%)	68 (41%)	**0.01**
Septic	115 (25%)	63 (21%)	52(31%)	**0.02**
Pancreatic fistula	44 (10%)	24 (8%)	20 (12%)	0.12
Bleeding	26 (6%)	15 (5%)	11 (7%)	0.28
Renal failure	18 (6%)	4 (1.5%)	14 (8%)	**<0.0001**
Reoperation	29 (6%)	19 (7%)	10 (6%)	0.3
				

*The cohort of PD patients included 293 patients; 173 aged <70 years, 120 ≥70 years

Kaplan-Meier survival analysis compared outcomes of ductal adenocarcinoma patients aged <70 years vs. ≥70 years. As depicted in Figure 1C patients aged 70 years or older had a lower median DSS in comparison to the younger cohort: **15 **months (95% CI: 12.5-26.5) vs. 20 months (95% CI: 16.1-38.3), respectively (p = 0.05). The estimated 1-, 2-, and 5-year DSS rates were 58% (SE: 3.1), 36% (SE: 3.6), and 23% (SE: 3.7) vs. 73% (SE: 4.8), 45% (SE: 4.2), and 27% (SE: 1.9) in the older cohort vs. the younger cohort, respectively.

## Discussion

Pancreatic surgery carries relatively high postoperative morbidity and long term outcomes for pancreatic cancer patients are relatively modest, yet complete resection for selected patients is the only curative therapeutic option. Nevertheless, well known increased risk for intra- and/or post-operative complications in the aged patients may create a dilemma for both the surgeon and the patient. Notwithstanding, a recent report showed that surgery is avoided in most patients diagnosed with resectable pancreatic neoplasm [19]. Another reported series demonstrated that due to age-based decisions elderly patients often receive less aggressive surgery, if any, as well as less systemic chemotherapy. Taken together these data, it is possible that age per-se plays a role in denying surgery from older patients diagnosed with resectable pancreatic malignancies; evidently such treatment patterns may result in inferior outcomes [20-24].

Most pancreatic tumors occur within the sixth or the seventh decade of life; however, a large proportion of patients are older [25,26]. Out of 460 evaluable patients with pancreatic neoplasm who had complete macroscopic resection at our institution over the last decade, 36% (n = 166) were older than 70 and 15.8% (n = 73) were 75 years or older. Moreover, the portion of patients aged 70 or more who had pancreatic surgery at our institution over the last decade has increased from 18% in 1996-2001 to 32.8% in 2002-2007. While this trend may simply reflect demographic changes (aging) in the population, it may also result from an increasing awareness that major surgical procedures can be performed safely in the elderly population in spite of significant comorbidities and/or reduced "physiological reserves" as compared to younger patients [9].

Reported data suggest increased perioperative morbidity and mortality risks following pancreatic surgery in older patients [15,27-29]; bathe et al [28] have shown an increased risk for immediate postoperative mortality (within 30 days) in patients older than 75 years, whereas Muscari and Riall [15,28] have demonstrated increased overall complications rate in pancreatic cancer octogenarians. The median age for the present elderly cohort was 75 years (range, 70-87) and more than half of the patients had ASA scores ≥ 3. Nevertheless, our data demonstrate that early postoperative outcomes for patients older than 70 years are comparable to previously published younger cohorts, which has also been reported by others [6,13,14,30,31]. Indeed, perioperative mortality rate was higher among elderly patients, 5.8% vs. 2.3% in the younger cohort; however, we find this rate acceptable considering the scale of the operation and the expected natural history of the disease without surgery. About 40% of the older patients cohort experienced postoperative complications; most of them fully recovered following conservative treatment.

As for long-term postoperative results, in a recent report, Riediger et al have shown that age was not an independent risk factor for pancreatic cancer-specific mortality when included in a multivariable Cox regression analysis [32]. This data supports previous findings reported by Fong, Richter, and others [13,33]. The current report with a five-year ductal adenocarcinoma-specific survival rate of 22% is similar to previous analyses including patients of all ages who underwent surgery for pancreatic adenocarcinoma in high volume centers [4,6,13,34]. Nevertheless, our data analysis identified age ≥70 years as an independent adverse prognostic factor for adenocarcinoma-specific survival with a hazard ratio of 1.64 (1.16-3.45).

Comparing the older group of patients (≥70 years) to the younger cohort, we found that older patients had a higher rate of malignant pathologies; this could be explained by selection bias rather than reflecting age-dependent biological differences. Adenocarcinoma histology, tumor size and grade were comparable, whereas pancreatic head tumors were more common in older patients. This may explain the higher incidence of jaundice as a presenting symptom among older patients. Interestingly, the rate of jaundice among the sub-group of patients who had PD for adenocarcinoma was higher among elderly individuals suggesting a more complex physiological explanation (20% vs. 35%; p = 0.001) attributed to a decreased functional reserve of the liver among these patients.

Adjuvant chemotherapy was significantly less frequently administered in the older as compared to the younger cohort (p = 0.003). In addition to various possible biological differences which may shorten the survival of elderly patients treated for pancreatic ductal adenocarcinoma less use of systemic therapy may also contribute to the higher rates of disease-specific mortality observed in these patients. While our data demonstrate that pancreatic cancer patients 70 years or older exhibit a lower five-year DSS compared to younger patients, it is pertinent that even in this age subset more than one third of the patients survived two years and 20% 5 years or more when treated with surgery. Taken together, these data, coupled with the acceptable rates of post operative morbidity, the equivalently low rates of post-operative mortality, and a reasonable length of hospital stay suggest that pancreatic surgery can be performed safely in older patients. This should encourage clinicians not to deny pancreatic resection from elderly patients, particularly those diagnosed with cancer. Comprehensive pre-operative assessment of surgical risks, careful pre-operative assessment, utilization of post-operative intensive care units, and rehabilitation services as needed are relevant to reducing overall peri-operative morbidity and mortality.

In summary, previous data suggest that elderly pancreatic cancer patients have worse outcomes; co-morbidities and decreased physiological reserves have historically reduced the enthusiasm of surgeons and oncologists to operate on these patients. Our analysis of retrospectively collected data from a single institution demonstrates that surgical resection of pancreatic neoplasms in the elderly can usually be performed safely with possible long term survival.

## Authors' contributions

GL participated in the design of the study, coordination, data collection and analysis, and drafting.

RS participated in data collection and analysis, and drafting. NL participated in drafting.

IN participated in drafting. FG participated in data collection and analysis and drafting.

MB participated in drafting. RN participated in drafting. JK participated in the design of the study and drafting. JMK participated in the design of the study and drafting.

All authors read and approved the final manuscript.

## Competing interests

The authors declare that they have no competing interests.
